# Genomic and Metabolomic Insights Into the Probiotic Potential of *Weissella viridescens*

**DOI:** 10.3390/biology15010063

**Published:** 2025-12-29

**Authors:** Shuwei Zhang, Ruiting Lan, Ruiqing Zhao, Ruoshi Wang, Liyun Liu, Jianguo Xu

**Affiliations:** 1 National Key Laboratory of Intelligent Tracking and Forecasting for Infectious Diseases, National Institute for Communicable Disease Control and Prevention, Chinese Center for Disease Control and Prevention, Beijing 102206, China; zhangshuweisc@163.com (S.Z.); stalker16@126.com (R.Z.); ruoshi_wang@163.com (R.W.); 2 School of Public Health, Nanjing Medical University, Nanjing 211166, China; 3 School of Biotechnology and Biomolecular Sciences, University of New South Wales, Sydney, NSW 2052, Australia; r.lan@unsw.edu.au; 4 Hebei Key Laboratory of Intractable Pathogens, Shijiazhuang Center for Disease Control and Prevention, Shijiazhuang 050011, China; 5 Department of Epidemiology, School of Public Health, Shanxi Medical University, Taiyuan 030001, China

**Keywords:** *Weissella viridescens*, complete genome, pangenome, metabolomics, probiotic traits, safety assessment

## Abstract

*Weissella viridescens* has been proposed as a probiotic candidate, but strain-level multi-omics evidence remains limited. The complete genome of the human-derived *W. viridescens* strain Wv2365 was sequenced through a hybrid assembly of Illumina and PacBio sequencing reads and compared with eight publicly available *W. viridescens* genomes. Pangenome analysis and functional annotation were performed, and metabolites were profiled by broadly targeted metabolomic analysis. In addition, the acid and bile tolerance, auto-aggregation and cell surface hydrophobicity, and antioxidant activity of the strain, as well as both in silico and phenotypic safety, were assessed. Wv2365 carries a single chromosome of 1.57 Mb with 41.3% G+C content. The species has an open pangenome with 803 core genes. Genomic and metabolomic features converged on carbohydrate and amino acid metabolism, including glycolysis/tricarboxylic acid (TCA) cycle and arginine pathways, and a carbohydrate-active enzyme (CAZyme) repertoire dominated by glycosyltransferases. In vitro, Wv2365 tolerated pH 3.0 and 0.3% bile, showed auto-aggregation, surface hydrophobicity, and 2,2-diphenyl-1-picrylhydrazyl (DPPH) and hydroxyl radical scavenging. The strain was susceptible to 10 antibiotics tested except for its intrinsic vancomycin non-susceptibility and was non-hemolytic and gelatinase negative. No acquired antimicrobial resistance or virulence genes were found in the genome. These findings indicate that *W. viridescens* Wv2365 is safe with probiotic traits relevant to gastrointestinal survival, colonization, and redox balance.

## Simple Summary

*Weissella viridescens* is a lactic acid bacterium that has been detected in fermented foods and the human intestinal tract, yet its probiotic potential remains poorly characterized at the strain level. In this study, we performed an integrated genomic, metabolomic, and phenotypic evaluation of a human-gut-derived strain, *W. viridescens* Wv2365. We found that this strain has metabolic characteristics related to carbohydrate and amino acid utilization, can tolerate acidic and bile conditions relevant to the gastrointestinal environment, and exhibits cell aggregation and antioxidant activity. Safety assessments did not indicate the presence of acquired antibiotic resistance or virulence-related traits. Overall, these findings suggest that Wv2365 is safe as a potential probiotic.

## 1. Introduction

*Weissella viridescens*, formerly classified as *Lactobacillus viridescens*, was reclassified into the genus *Weissella* in 1993 [1]. It is a small, tapered, rod-shaped lactic acid bacterium (LAB) [2]. *W. viridescens* is frequently recovered from fermented foods—including dry-fermented sausages, Thai “Nham,” soybean pastes, and spontaneously fermented vegetables [2]—and it has also been detected in human-associated niches such as the vaginal and intestinal microbiota [3]. Although the genus *Weissella* is less studied than *Lactobacillus* or *Bifidobacterium*, accumulating evidence points to a promising probiotic profile for *W. viridescens*, with strains showing antimicrobial and anti-inflammatory activities and producing bioactive metabolites (e.g., organic acids and exopolysaccharides) [4,5], highlighting its potential for health and fermented-food biotechnology.

Probiotics are defined as live microorganisms that, when administered in adequate amounts, confer health benefits on the host [6]. LAB are among the principal sources of candidate probiotic strains because of their long history of safe use in foods and their well-documented beneficial gastrointestinal and immunomodulatory effects [7]. Within this group, *Lacticaseibacillus rhamnosus* GG (LGG) is one of the most extensively studied probiotic strains and is commonly used as a reference strain in probiotic research [8,9]. Probiotic functionality, however, is highly strain-specific, and current guidelines emphasize that each candidate strain should be evaluated individually for efficacy and safety. Contemporary probiotic research has therefore moved beyond purely phenotype-based screening and increasingly relies on strain-level genomic and other multi-omics characterization, together with in vitro and in vivo assays, to define probiotic potential, elucidate mechanisms of action, and assess safety in line with World Health Organization/Food and Agriculture Organization (WHO/FAO) and European Food Safety Authority (EFSA) recommendations [10,11].

At the genus level, core- and pangenome studies of *Weissella cibaria* have delineated a LAB-like core proteome broadly similar to other lactic acid bacteria [12], and multi-species comparisons have shown phylogenies that respect species boundaries while revealing within-species diversity likely linked to niche adaptation [13]. In these analyses, acquired antimicrobial resistance and classical virulence genes were generally absent in *Weissella* spp. However, genomic and pangenomic investigations have so far focused mainly on food-derived *Weissella* species, and *W. viridescens* remains comparatively under-characterized. For *W. viridescens*, only a few genomes have been sequenced, and systematic multi-omics studies linking genomic features, extracellular metabolic output, and probiotic traits are lacking. Human-gut-derived *W. viridescens* strains are rarely described, and it is unclear whether they share the same functional characteristics as food isolates or display host-adapted properties relevant to intestinal colonization and metabolic modulation.

We previously showed that a human-derived *W. viridescens* strain, Wv2365, isolated from the gut of a healthy adult in China, ameliorated metabolic dysfunction-associated steatotic liver disease (MASLD) in rats [14], supporting its probiotic promise at the organismal level. However, the strain-level genomic basis and metabolomic profile underlying these effects remain unknown. Here, we present the complete genome of *W. viridescens* Wv2365 and compare it with publicly available *W. viridescens* genomes. We further integrated genomic analysis of KEGG pathways with metabolomics of the culture supernatant to better understand its metabolic profile. Finally, we benchmarked key in vitro probiotic traits (acid and bile tolerance, auto-aggregation and hydrophobicity, antioxidant capacities) and evaluate safety using in silico screening and standard phenotypic assays.

## 2. Materials and Methods

### 2.1. Bacterial Strain and Culture

*W. viridescens* Wv2365 is a human-gut-derived strain that was previously isolated and characterized as described in Zhang et al. [14]. The strain used in this study was obtained from the China General Microbiological Culture Collection Center (CGMCC; accession number CGMCC 27140) and stored at −80 °C. Unless otherwise stated, Wv2365 was cultured at 37 °C in a 5% CO_2_ atmosphere for 18–24 h on De Man, Rogosa, and Sharpe (MRS) agar or broth (Oxoid, Lenexa, KS, USA) supplemented with 5% (*v*/*v*) defibrinated sheep blood.

### 2.2. Whole-Genome Sequencing, Assembly, and Annotation

Genomic DNA was extracted from overnight culture using a commercial bacterial DNA kit (Tiangen Biotech Co., Ltd., Beijing, China). DNA quality was assessed by NanoDrop, Qubit, and 1% agarose gel electrophoresis. Paired short reads (Illumina HiSeq, Illumina, Inc., San Diego, CA, USA) and long reads (PacBio Sequel, Pacific Biosciences of California, Inc., Menlo Park, CA, USA) were generated (Majorbio, Shanghai, China). Illumina reads were quality-trimmed with Trimmomatic; PacBio subreads were processed with SMRT Link. Hybrid *de novo* assemblies were produced with Unicycler [15]. Assemblies were polished with Pilon (short-read polishing) and Racon (long-read polishing). Possible circularization of contigs was assessed, not assumed, using Circlator and Unicycler diagnostics; when a circular replicon was indicated, sequences were rotated to place *dnaA* at the origin. Plasmid candidates were screened with PlasmidFinder. Assembly statistics (N50, coverage, GC%) were computed with QUAST. Gene prediction used Prokka (CDSs, tRNAs, rRNAs, sRNAs). Coding density was calculated as the total length of all annotated protein-coding sequences divided by the chromosomal genome size, based on Prokka-generated genome annotations. Functional annotation was performed using eggNOG-mapper for Clusters of Orthologous Groups (COGs) and Gene Ontology (GO) terms, and KofamKOALA/KEGG Mapper for Kyoto Encyclopedia of Genes and Genomes (KEGG) orthologs and pathways, with BLAST+ (v2.2.28) used for manual curation when necessary [16]. Carbohydrate-active enzymes (CAZymes) were identified via BLASTP and HMMER searches against the CAZy database [17]. Circular genome maps (CDS tracks, RNA features, COG classes, GC content/skew) were produced with CGView. Raw reads have been deposited under BioProject PRJNA1250212. The complete genome was submitted to GenBank under accession number CP187404.

### 2.3. Pangenome and Comparative Genomics

All publicly available *W. viridescens* genomes with scaffold-level or higher assemblies (retrieved from NCBI on 30 September 2025; accession numbers listed in Supplementary Table S1) were re-annotated using Prokka v1.14.6 for consistent comparative analysis with Wv2365. Orthologous clustering [18] and pangenome analysis were performed with Roary v3.13.0 on the Prokka GFF3 outputs a BLASTp identity threshold of 90% (-i 90) and a core gene definition threshold of 99% (-cd 99), with MAFFT v7.520 for alignment [19]. The presence–absence matrix generated by Roary was used to classify gene clusters based on their distribution across the nine genomes. Genes present in all nine strains were defined as core genes. Genes present in eight strains were classified as soft-core genes. Genes detected in two to seven strains were designated as shell genes, whereas those present in only a single strain were considered cloud genes (strain-specific genes). The core gene alignment was used to construct a maximum-likelihood phylogenetic tree with FastTree v2.1.11 (Jukes–Cantor model, 1000 bootstraps) [20]. Pan and core genome accumulation curves were also generated from the presence/absence matrix to evaluate genome openness and strain-specific gene diversity. Single nucleotide variants (SNVs) were categorized according to whether they were located in core or accessory genes based on the Roary pangenome matrix.

### 2.4. Metabolomic Profiling of W. viridescens Wv2365 Supernatant

Wv2365 was cultured in MRS liquid broth at 37 °C in a 5% CO_2_ atmosphere for 24 h to reach the stationary phase, and supernatants were collected for metabolomic analysis. An uninoculated MRS broth processed in parallel under identical conditions served as a background control. Supernatants were collected by centrifugation (4000× *g*, 10 min, 4 °C) and 0.22 µm-filtered. Metabolites were extracted with cold methanol/acetonitrile (1:1, *v*/*v*), followed by vortexing and centrifugation to remove proteins. The supernatants were dried in a vacuum centrifuge and reconstituted in 100 μL acetonitrile/water (1:1, *v*/*v*) prior to LC–MS analysis. Chromatography was performed on an Agilent 1290 UHPLC with HILIC and C18 separations, coupled to an AB Sciex 6500+ QTRAP (Sciex, Framingham, MA, USA) operated in multiple-reaction monitoring (MRM) for broad targeted detection. Peak areas were integrated in Analyst/MultiQuant with internal standard normalization; where available, external standard curves were used for absolute quantification. To distinguish metabolites attributable to Wv2365 from medium-derived signals, features detected in the MRS control were used for background correction. Only metabolites detected at higher levels in the Wv2365 supernatants than in the uninoculated MRS control, after background correction, were retained for downstream analyses, reflecting extracellular metabolites produced or accumulated during growth. Quality control samples were injected at regular intervals to monitor analytical stability. Detected features were mapped to KEGG for pathway assignment [21], enrichment analysis used right-tailed Fisher’s exact tests with multiple-testing correction, and results were summarized as bubble plots consistent with the main figures.

### 2.5. In Vitro Probiotic Trait Assays

#### 2.5.1. Acid and Bile Salt Tolerance

Acid and bile salt tolerance were assessed with minor modifications to published protocols [22]. Logarithmic-phase *W. viridescens* cells were inoculated into MRS broth at an initial concentration of 10^7^ CFU/mL. For acid tolerance, the test group was incubated in MRS adjusted to pH 3.0, while the control group was maintained in MRS (pH 6.3, unadjusted); cultures were incubated at 37 °C under 5% CO_2_ for 3 h [23]. For bile salt tolerance, cells were inoculated into MRS containing 0.3% (*w*/*v*) bile salts (Solarbio Life Sciences, Beijing, China), with bile-free MRS as the control; incubation was at 37 °C, 5% CO_2_ for 4 h. In both assays, viable counts before and after incubation (N_0_ and N_1_) were determined by serial dilution and plate counting. Survival rate(%) = (lgCFU N_1_/lgCFU N_0_) × 100%. *Lacticaseibacillus rhamnosus* GG (LGG, ATCC 53105) served as the positive control [24].

#### 2.5.2. Auto-Aggregation and Hydrophobicity Assays

For auto-aggregation, freshly cultured cells were suspended in PBS (Invitrogen, Carlsbad, CA, USA) and adjusted to McFarland 1.0 (OD_0_). After static incubation at 37 °C and 5% CO_2_ for 24 h, the optical density of the supernatant (OD_1_) was measured. Auto-aggregation (%) = [1 − (OD_1_/OD_0_)] × 100%. For cell-surface hydrophobicity, cells were suspended in 0.85% (*w*/*v*) NaCl (bioMérieux, Marcy-l’Étoile, France) and adjusted to 1.0 (OD_0_). Xylene was added at a 1:1 (*v*/*v*) ratio (2 mL cell suspension: 2 mL xylene; Sinopharm Chemical Reagent Co., Ltd., Shanghai, China), the mixture was vortexed thoroughly, and then statically incubated at 37 °C, 5% CO_2_ for 1 h. After phase separation, the optical density of the aqueous phase (OD_1_) was recorded [22]. Hydrophobicity (%) = [1 − (OD_1_/OD_0_)] × 100%. In both assays, LGG was used as the positive control.

#### 2.5.3. Antioxidant Activity Assays

Fermentation broths were prepared by culturing strains (10^8^ CFU/mL) in MRS at 37 °C, 220 rpm for 24 h; supernatants were obtained by centrifugation (4000× *g*, 10 min, 4 °C) and filtration (0.22 μm). For 2,2-diphenyl-1-picrylhydrazyl (DPPH) radical scavenging [25], reaction mixtures contained 2 mL of 0.04 mmol/L DPPH (Sigma-Aldrich, St. Louis, MO, USA) in ethanol and 2 mL of sample supernatant; after incubation at 37 °C, 5% CO_2_ for 30 min, absorbance was read at 517 nm. For hydroxyl radical scavenging, total reaction volume was 5 mL, comprising 1 mL 2.5 mmol/L o-phenanthroline (Solarbio Life Sciences, Beijing, China), 1 mL 2.5 mmol/L FeSO_4_, 1 mL sample supernatant, 1 mL 20 mmol/L H_2_O_2_, and 1 mL deionized water; after 30 min at 37 °C, absorbance was recorded at 517 nm. Scavenging (%) = [1 − (OD_sample − OD_blank)/OD_control] × 100. LGG supernatant was used as the positive control.

### 2.6. Safety Evaluation of W. viridescens Wv2365

#### 2.6.1. In Silico Genomic Safety Assessment

For genome-based safety screening (in silico), putative virulence and host-interaction factors were annotated against the Virulence Factor Database (VFDB) [26] and the Pathogen–Host Interaction Database (PHI-base) using DIAMOND blastp with an E-value cutoff of 1 × 10^−5^. Potential acquired antibiotic resistance genes (ARGs) were queried using ResFinder [27] and the Comprehensive Antibiotic Resistance Database (CARD) [28], with searches performed under both perfect and strict criteria. Annotations were summarized by functional categories to inform downstream phenotypic testing.

#### 2.6.2. In Vitro Phenotypic Safety Assessment

To verify genomic predictions, antimicrobial susceptibility testing followed both the Clinical and Laboratory Standards Institute (CLSI) M45 guideline [29] and the EFSA recommendations for safety assessment of LAB. Because no species-specific minimum inhibitory concentration (MIC) breakpoints are available for *Weissella* in either guideline, the antimicrobial panel was selected based on EFSA- and CLSI-recommended antibiotics for LAB [30]. MIC interpretation was performed strictly according to the CLSI M45 criteria for *Leuconostoc* spp., the closest phylogenetic relative of *Weissella* [2]. Susceptibility criteria for *Lactobacillus* spp. were additionally consulted as contextual reference values. Bacterial cultures were adjusted to a 0.5 McFarland turbidity standard and inoculated onto cation-adjusted Mueller–Hinton agar supplemented with 2–5% lysed horse blood (CAMHB-LHB; Oxoid, Basingstoke, UK), as required by CLSI M45 for fastidious Gram-positive bacteria. MICs for 10 antibiotics (penicillin, ampicillin, tetracycline, chloramphenicol, vancomycin, linezolid, meropenem, levofloxacin, erythromycin, and clindamycin) were determined using E-test concentration-gradient strips (Liofilchem, Roseto degli Abruzzi, Italy). Plates were incubated at 35 ± 2 °C in ambient air for 20–24 h, and MIC values were interpreted strictly according to CLSI M45. *Streptococcus pneumoniae* ATCC 49619 served as the quality-control strain.

Hemolysis was assessed by spotting 10 µL of ~10^8^ CFU/mL suspension onto BHI + 5% (*v*/*v*) defibrinated sheep blood, incubated 24 h at 37 °C, 5% CO_2_, and classified as α/β/γ based on colony halos [31]. Gelatinase activity was tested on BHI + 3% gelatin (Aladdin Biochemical Technology Co., Ltd., Shanghai, China); after 72 h under the same conditions, plates were flooded with saturated (NH_4_)_2_SO_4_ and examined for clearance zones indicating positivity [32]. *Staphylococcus aureus* ATCC 25923 was used as the positive control for the gelatinase assay.

### 2.7. Statistics

Analyses were conducted in R 4.4.2. Two group comparisons used two-tailed t-tests; for multiple comparisons within a panel, Tukey’s post hoc tests were applied as appropriate. Significance was set at *p* < 0.05. All assays were performed using at least three independent biological experiments, each comprising at least three technical replicates. Data were reported as mean ± SD.

## 3. Results

### 3.1. Genome Features of W. Viridescens Wv2365

**Figure 1:**
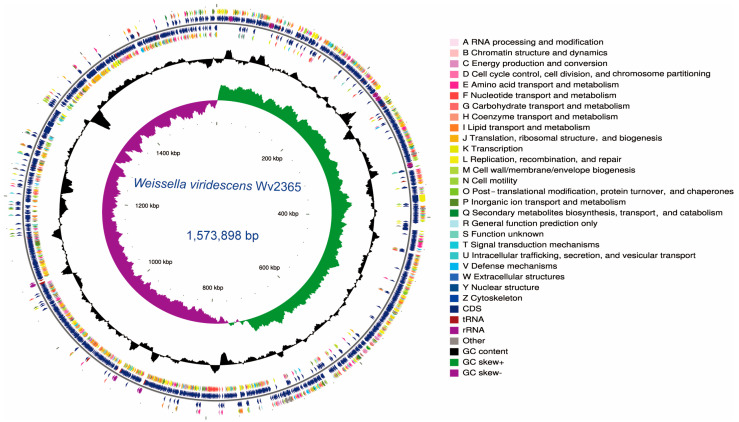
Circular genome map of *W. viridescens* Wv2365. The outer rings display coding sequences colored by COG functional categories, followed by the locations of rRNA and tRNA genes. Inner rings represent GC content and GC skew, highlighting local compositional variation and strand bias along the chromosome.

The genome of *W*. *viridescens* Wv2365 was completely sequenced using Illumina and PacBio sequencing, with a mean sequencing depth of 322.7 (Figure 1). The genome consisted of a single circular chromosome of 1,573,898 bp with a GC content of 41.3% and contains no plasmids. There are 1455 protein-coding sequences (average length 944.6 bp; coding density 87.3%), as well as 77 tRNA genes, 25 rRNA genes (8 × 16S, 8 × 23S, 9 × 5S), and 18 sRNA loci. As shown in Figure 1, the GC-content profile remains relatively stable across the chromosome without large regions of pronounced deviations, suggesting the absence of major compositionally distinct segments. The GC-skew curve exhibits a single polarity switch, consistent with a typical bidirectional replication pattern. The distribution of COG-classified CDSs does not reveal any extensive functional clustering, indicating no obvious large-scale structural irregularities.

### 3.2. Pangenome Analysis of the W. viridescens Dataset

**Figure 2:**
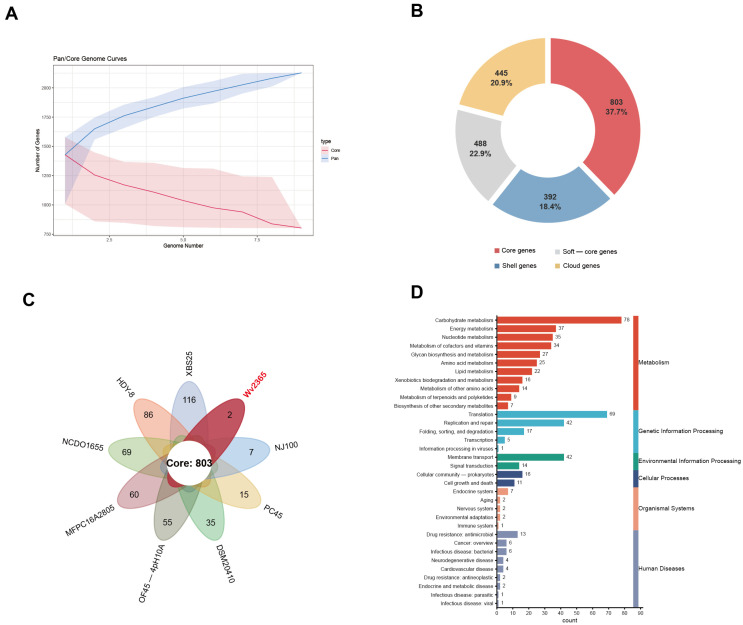
Pangenome analysis of *W. viridescens* strains. (**A**) Pan/core curves. The *x*-axis represents the number of genomes sequentially added to the analysis, and the *y*-axis indicates the number of gene clusters. (**B**) Genome partitioning into core genes (present in all nine strains), soft-core genes (present in eight strains), shell genes (present in two to seven strains), and cloud genes (present in only one strain). Counts and percentages for each category are shown. (**C**) Flower plot with 803 core genes shared by all strains. (**D**) KEGG functional distribution of core genes. Note that for the KEGG “Energy metabolism” category, most of the Wv2365 genes belonged to the subcategory oxidative phosphorylation.

Nine *W. viridescens* genomes, including Wv2365 and eight publicly available strains (Table S1), were used to determine the pan and core genomes. Pairwise average nucleotide identity (ANI) analysis showed high genomic similarity (>98%) among the nine strains (Figure S1A). The pangenome has 2128 genes with an open pangenome (Figure 2A). There were 803 core genes (37.7%), 392 soft-core (18.4%), 488 shell (22.9%), and 445 cloud (20.9%) (Figure 2B, Table S2). Strain-specific genes varied from 2 to 116. Wv2365 harbors only two strain-specific genes encoding a chromosome partitioning protein (*Smc*) and a putative multidrug export ATP-binding/permease protein (Figure 2C); the UpSet plot illustrates broad gene intersections across strains, indicating a conserved genomic backbone (Figure S1B). After KEGG functional annotation of the 803 core genes, most were assigned to basic metabolic pathways (Figure 2D), mainly involving carbohydrate metabolism, energy metabolism, nucleotide metabolism, and the metabolism of cofactors and vitamins.

### 3.3. Phylogenetic Placement and SNV Comparison of Wv2365

**Figure 3:**
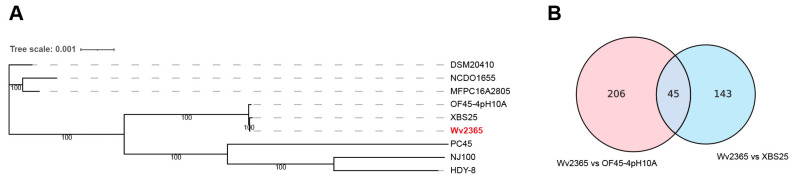
Phylogenetic relationship and SNV comparison of Wv2365 and related *W. viridescens* strains. (**A**) Maximum-likelihood phylogenetic tree based on the core gene alignment of nine W. viridescens genomes. Bootstrap support values (%) are shown on internal nodes, and the scale bar indicates substitutions per site. Dotted horizontal lines are provided to assist alignment of strain names with branches for visualization and Wv2365 was colored in red to highlight the studied strain. (**B**) Venn diagram showing shared and strain-specific SNVs identified using Wv2365 as the reference genome for pairwise comparisons with XBS25 and OF45-4pH10A.

The Wv2365 genome was compared with eight publicly available *W. viridescens* genomes. A core gene phylogeny showed that Wv2365 clustered most closely with strains XBS25 and OF45-4pH10A, whereas the remaining six strains formed distinct lineages (Figure 3A). Using Wv2365 as the reference, a total of 394 non-redundant SNVs were identified across the pairwise comparisons. Of these, 206 SNVs were unique to the comparison with OF45-4pH10A, 143 were unique to the comparison with XBS25, and 45 SNVs were shared by both comparisons (Figure 3B). Among the 394 SNVs, 182 (46.2%) were located in core genes present in all nine genomes, whereas 209 (53.0%) were located in accessory genes. Three SNVs were located outside annotated protein-coding genes. A complete list of SNVs is provided in Supplementary Table S3.

### 3.4. Functional Genome Annotation of W. Viridescens Wv2365

**Figure 4:**
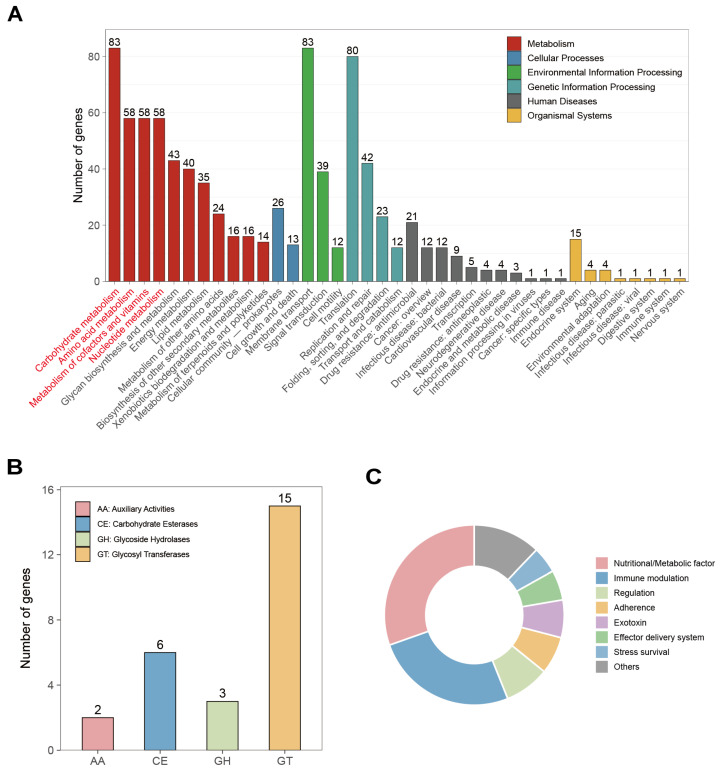
Functional genome annotation of *W. viridescens* Wv2365. (**A**) KEGG functional categorization of coding genes. The four major KEGG metabolic categories with the highest gene proportions (*x*-axis labels) were highlighted in red. (**B**) Distribution of carbohydrate-active enzymes (CAZymes). (**C**) Functional categorization of predicted virulence-associated genes in Wv2365 based on VFDB annotation. A complete list of the identified genes, including sequence identity, coverage, E-values, and VF categories, is provided in Supplementary Table S4.

Using the KEGG database, 946 (65.0%) of the 1455 predicted protein-coding genes of *W. viridescens* Wv2365 were functionally annotated. In addition, 1216 (83.6%) and 1047 (72.0%) genes were assigned to the COG categories and GO terms, respectively, as shown in Supplementary Figure S2A,B. KEGG analysis identified major pathways associated with carbohydrate metabolism (83 genes), amino acid metabolism (58 genes), metabolism of cofactors and vitamins (58 genes), and nucleotide metabolism (58 genes) (Figure 4A). At the KEGG level-3 classification, carbohydrate metabolism mainly comprised glycolysis/gluconeogenesis, pyruvate metabolism, and the pentose phosphate pathway, while amino acid metabolism included lysine, alanine/aspartate/glutamate, and cysteine/methionine pathways (Figure S2C). Annotation of CAZymes revealed a total of 26 CAZymes, among which glycosyltransferases (GTs) were the most abundant, followed by carbohydrate esterases (CEs) and glycoside hydrolases (GHs) (Figure 4B).

VFDB analysis identified 148 virulence-associated genes in the *W. viridescens* Wv2365 genome (Table S4), most of which were classified into nutritional and metabolic factors (45), immune modulation (38), and adherence (10) (Figure 4C). Using PHI-base classification, most of the identified virulence-associated genes corresponded to “reduced virulence” or “unaffected pathogenicity” classes (Supplementary Figure S2D). No acquired antimicrobial resistance genes were detected using ResFinder. However, using CARD, two glycopeptide-related homologs were identified as vancomycin resistance genes, *vanT*-like and *vanY*-like, with sequence identities of 33.6% and 30.7%, respectively.

### 3.5. Metabolomic Profiling of W. viridescens Wv2365 Culture Supernatant

**Figure 5:**
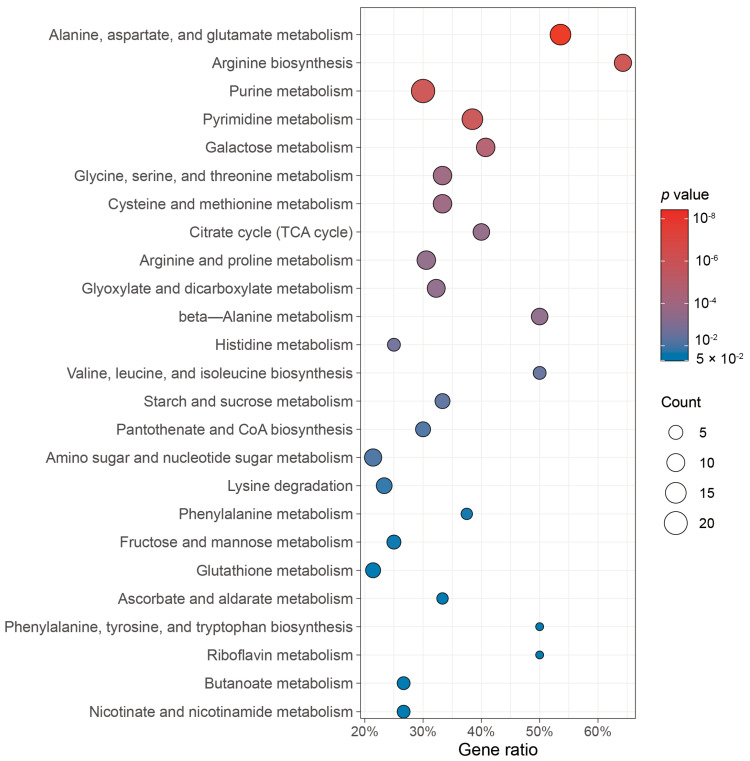
KEGG pathway enrichment of metabolites produced by *W. viridescens* Wv2365. Bubble size represents the number of identified metabolites enriched in each pathway, and color coded heatmap indicates the enrichment *p* value. Colors are mapped to −log10 (*p*) values for visualization. Gene ratio refers to the proportion of identified metabolites mapped to a given pathway relative to the total number of annotated metabolites in that pathway, expressed as a percentage. The *p* values shown are raw *p* values derived from Fisher’s exact test; the corresponding adjusted *p* values (FDR) controlling for multiple testing are provided in Supplementary Table S6.

Metabolomic analysis of the Wv2365 culture supernatant identified 251 metabolites, primarily carbohydrates, organic acids, amino acids, and nucleotides (Table S5). KEGG pathway analysis of the metabolites found significant enrichment of amino acid metabolism (e.g., alanine, aspartate, and glutamate metabolism; arginine biosynthesis), nucleotide metabolism (e.g., purine metabolism), and carbohydrate metabolism (e.g., galactose metabolism and the TCA cycle), suggesting broad metabolic activity of Wv2365 (Figure 5).

### 3.6. In Vitro Probiotic Traits of W. viridescens Wv2365

**Figure 6:**
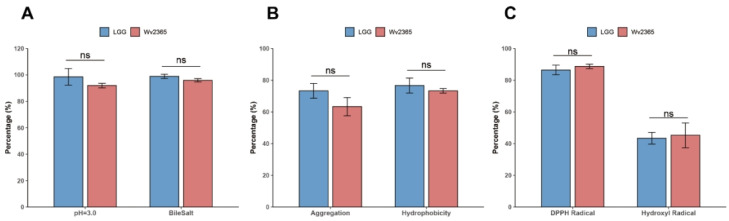
In vitro probiotic characteristics of *W. viridescens* Wv2365. (**A**) Survival under acidic conditions (pH 3.0) and in the presence of 0.3% bile salts. (**B**) Auto-aggregation ability and cell surface hydrophobicity. (**C**) Radical scavenging activity against DPPH and hydroxyl radicals. LGG was used as the reference probiotic strain for comparison. Data are presented as mean ± SD (*n* ≥ 3 independent biological experiments). ns indicates no statistically significant difference.

*W. viridescens* Wv2365 was evaluated for its in vitro probiotic properties. As shown in Figure 6, the strain demonstrated high survival rates under simulated gastrointestinal stress conditions (pH 3.0 and 0.3% bile salts), strong adhesive potential characterized by auto-aggregation and cell-surface hydrophobicity, and considerable antioxidant capacity in scavenging DPPH and hydroxyl radicals. Overall, these probiotic traits were comparable to those of LGG, with no significant differences observed (*p* > 0.05).

### 3.7. Safety Evaluation of Results W. viridescens Wv2365

*W. viridescens* Wv2365 was tested against 10 antibiotics and showed sensitivity to all except vancomycin (Table 1).

**Figure 7:**
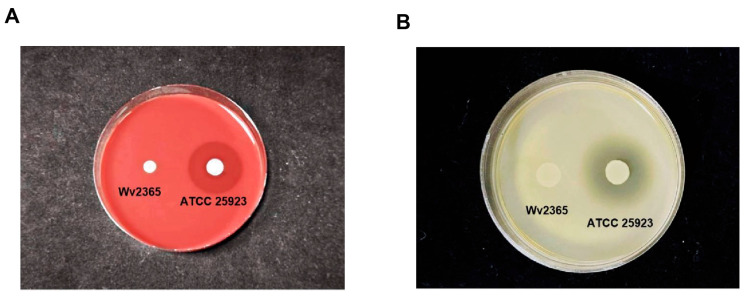
Hemolytic and gelatinase activity of *W. viridescens* Wv2365: (**A**) blood agar hemolysis (γ-hemolysis); (**B**) gelatinase assay.

The safety characteristics of the strain were further examined through hemolysis and gelatinase assays. As shown in Figure 7, no hemolytic zones were observed on blood agar, and no clear zones of gelatin hydrolysis were detected on gelatin agar, demonstrating that Wv2365 lacked hemolytic and gelatinase activities, respectively.

### 3.8. Overview of Probiotic Traits of W. viridescens Wv2365 as Supported by Multi-Omics Evidence

**Figure 8:**
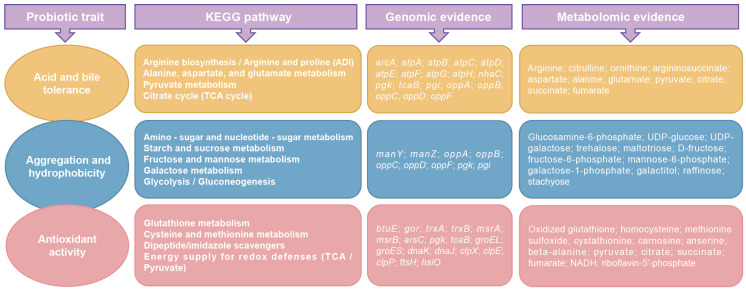
The figure summarizes phenotypic properties as probiotic traits and the corresponding KEGG pathways that are associated with these traits and genomic and metabolic evidence. The KEGG pathways represent overlapping functional categories identified from both genomic and metabolomic analyses, with the corresponding genes and metabolites shown.

To provide an integrated view of the multi-omics data, we summarized the probiotic traits of Wv2365 together with the KEGG pathways enriched from both the genomic and metabolomic analyses (Figure 8). As shown in Table S7, genes associated with acid and bile stress responses (e.g., *atp* operon, *arc* genes, *nhaC* antiporter), adhesion-related transport systems (such as *manY*/*Z* and *oppA–F*), and oxidative or redox-associated functions (including *trxA*/*B*, *gor*, *gshA*/*B*, and *msrA*/*B*) were identified in the genome. These gene categories correspond to KEGG functional pathways that were also represented in the metabolomic dataset, where metabolites belonging to carbohydrate-utilization, amino acid metabolism, and redox-related pathways were detected. Collectively, these KEGG pathways reflect overlapping functional categories independently supported by genomic annotation and metabolite enrichment, providing a consolidated multi-omics overview of the probiotic traits observed in vitro.

## 4. Discussion

Our previous study found that *W. viridescens* strain Wv2365 can prevent MASLD in rats [14] and thus is a potential probiotic. Although Wv2365 was isolated from a human subject in China, the original source or its evolutionary origin of the strain is unknown, as *Weissella* species are widely distributed across diverse food matrices and geographical regions. To further assess its probiotic potential, we performed genomic, metabolomic, and phenotypic analyses of the strain. We obtained the complete genome of Wv2365 and determined the core genome of *W. viridescens* based on nine available genomes. Wv2365 shared a core of 803 genes with only two strain-specific genes. Integrative genomic and metabolomic analyses found that the main metabolic capacity is centered on carbohydrate and amino acid metabolism. Phenotypically the strain is acid and bile tolerant, auto-aggregative/surface hydrophobic, and antioxidant capable. Wv2365 also displayed a favorable safety profile, with no classical virulence determinants identified in the genome and a generally susceptible antimicrobial profile; the observed vancomycin non-susceptibility is consistent with an intrinsic trait reported for lactic acid bacteria. Compared with many food-derived *Weissella* and classical lactic acid probiotic strains, Wv2365 is distinguished by its human-gut origin and the previously demonstrated in vivo efficacy against MASLD, while exhibiting in vitro probiotic traits that are broadly comparable to those of well-characterized strains. Therefore, Wv2365 is a probiotic candidate with potential as a general probiotic or disease-targeted probiotic.

### 4.1. Conserved Genomic Architecture with Strain-Level Diversification in W. viridescens

The pangenome analysis provided a comparative framework for evaluating the genomic features of Wv2365 within *W. viridescens*. The identification of a conserved core genome indicates that essential metabolic and cellular functions are largely shared across strains, whereas the presence of a flexible accessory gene repertoire is consistent with variability in traits associated with environmental adaptation [33,34]. In this context, the limited number of strain-specific genes in Wv2365 suggests that its gene content is closely aligned with the shared genomic background represented by the currently available strains. Beyond gene content, the distribution of SNVs across both core and accessory genes indicates that genomic differentiation within *W. viridescens* also involves sequence-level variation in conserved loci. Although many variants in core genes are unlikely to have strong functional consequences individually, non-synonymous substitutions affecting metabolic, stress-response, or regulatory pathways may contribute to incremental functional differences when accumulated across multiple loci. These features are consistent with a scenario in which *W. viridescens* strains retain a common genomic framework while undergoing strain-level diversification shaped by both accessory gene composition and accumulated sequence variation.

### 4.2. Genomic and Metabolomic Evidence for Efficient Carbohydrate Utilization by Wv2365

The Wv2365 genome shows a pronounced capacity for carbohydrate and amino acid metabolism, which is concordant with the metabolomic profile dominated by glycolysis, the TCA cycle, and amino acid pathways. CAZymes are a class of enzymes that catalyze the degradation, modification, or synthesis of complex carbohydrates and are central to microbial carbohydrate processing [17]. CAZymes are widespread in lactic acid bacteria, with glycosyltransferases (GTs) frequently represented [35]. We found that Wv2365 encodes 26 CAZymes. The predicted GH13 (α-amylase) and GH3 (β-glucosidase) suggest that the strain has the capacity to hydrolyze starch- and cellulose-derived polysaccharides, which is consistent with the detection of maltotriose and d-cellobiose as carbohydrate degradation intermediates in culture supernatants. The predicted GT4-family enzyme (trehalose-6-phosphate synthase) indicates a potential for trehalose metabolism. These features are consistent with previous genome analysis of *W. cibaria* YRK005 and *W. confusa* CCK931 strains, both of which encode a GH13_30 α-glucosidase that hydrolyzes maltotriose [36]. Therefore, Wv2365 is genomically equipped for efficient processing of starch-derived and plant-associated oligosaccharides. These traits likely contribute to its probiotic potential.

### 4.3. Extracellular Metabolite Profiles Link Active Metabolism to Functional Traits in Wv2365

Metabolomic profiling of the Wv2365 culture supernatants yielded concordant features of alanine–aspartate–glutamate metabolism, arginine biosynthesis, and glycolysis-to-TCA flux, alongside activity in galactose and fructose/mannose pathways and purine/pyrimidine turnover. These extracellular metabolite profiles reflect active pathways and genome-inferred functions in LAB [37]. The accumulation of UDP-glucose/UDP-galactose links directly to cell-wall and surface-polysaccharide biosynthesis, offering a mechanistic bridge to the observed aggregation and hydrophobicity [38,39]. In addition, several organic acids were detected in the Wv2365 culture supernatant, which may contribute to ecological competitiveness and could be relevant to antimicrobial traits commonly reported in LAB [40]. However, no putative bacteriocin genes were detected in the Wv2365 genome.

### 4.4. Integrated Genomic and Metabolomic Evidence Underpin Probiotic-Relevant Phenotypes

Viewed together, these probiotic-relevant phenotypes can be mechanistically interpreted in the context of integrated genomic features and metabolomic signatures. The acid and bile survival in Wv2365 is likely mediated by a multi-component system comprising the arginine deiminase pathway (*arc*), the F_0_F_1_-type ATP synthase operon (*atpA*–*H*), and NhaC-type cation–proton antiporters, which together sustain intracellular pH and ion homeostasis [41,42], with arginine-cycle intermediates (citrulline, ornithine) and central organic acids corroborating pathway activity under acid and bile stress [43,44]. Aggregation and hydrophobicity were associated with carbohydrate-utilization and transport pathways (amino-sugar/nucleotide-sugar, starch–sucrose, fructose–mannose, galactose), including the mannose-type PTS (an intact *manY*/*Z* operon in the Wv2365 genome) and the oligopeptide transporter (an intact *oppA–F* operon in the Wv2365 genome), alongside sugar–phosphate intermediates such as G6P, UDP-glucose, and UDP-galactose, which together suggest a possible link between carbohydrate metabolism and cell-envelope composition, potentially influencing surface properties such as aggregation and hydrophobicity [45,46,47,48]. Antioxidant capacity may be supported by a thiol-redox and proteostasis network encoded in the Wv2365 genome (including *btuE*, *trxA*/*B*, *gor*, *gshA*, *msrA*/*B*, and chaperone systems such as *groEL*/*ES*, *dnaK*/*J*, *clpX*/*E*/*P*, *ftsH*, and *hslO*), together with the accumulation of extracellular redox-active metabolites (e.g., oxidized glutathione and NADH) and the observed DPPH- and OH-scavenging activities [49,50,51,52].

### 4.5. In Vitro Phenotypic Evidence Supports the Probiotic Potential of Wv2365

The in vitro assays further substantiated the probiotic potential inferred from the multi-omics analysis. Gastric acid and bile salts are recognized as two major challenges for the survival of orally administered probiotics. Strains capable of withstanding low pH and high bile concentrations are more likely to survive gastrointestinal transit and exert beneficial effects within the host intestine. Consistent with commonly applied in vitro screening strategies, LAB isolated from traditional fermented vegetables have been shown to retain substantial viability under acidic conditions and tolerance to bile salts [23]. Within this broader context of LAB screening, this observation is consistent with earlier reports that *W. confusa* 31 and several *W. viridescens* isolates possess inherent acid–bile resistance [53,54], suggesting that such tolerance may be a genus-level trait. Cell surface hydrophobicity and auto-aggregation are commonly used as preliminary, indirect surface-trait assays in probiotic screening [55], and Wv2365 exhibited levels comparable to those reported for other well-characterized LAB. For comparison, *Limosilactobacillus reuteri* isolates K7 and K14, together with other LAB and *bifidobacteria*, exhibited notable auto-aggregation and cell surface hydrophobicity phenotypes [56]. Finally, Wv2365 exhibited marked in vitro antioxidant activity. Similar antioxidant-related phenotypes have been reported in several LAB, including *Lactiplantibacillus plantarum* CCMA 0743, *Lactobacillus delbrueckii* subsp. *bulgaricus* ATCC 11842, and *Streptococcus thermophilus*, indicating that antioxidant capacity and oxidative stress tolerance are recurring functional traits among diverse LAB [57].

### 4.6. Integrated Genome- and Phenotype-Based Safety Assessment of Wv2365

In addition to functional probiotic traits, integrated genomic and phenotypic analyses provided important insights into the safety profile of *W. viridescens* Wv2365. Although multiple genes were annotated as virulence-associated based on VFDB homology searches (Table S4), these genes largely correspond to conserved functions commonly identified in *Weissella* genomes rather than bona fide virulence determinants. Similar observations have been reported in comparative genomic analyses of *Weissella* species. For example, putative virulence-associated genes such as *tufA* (encoding elongation factor Tu), *lisR* (encoding two-component response regulator), and enzymes involved in carbohydrate metabolism (e.g., *hasC*, encoding DP-glucose pyrophosphorylase, and *SMU_322c*, encoding glucose-1-phosphate uridylyltransferase) were identified in *W. hellenica* 0916-4-2 and *W. cibaria* UTNGt21O and were interpreted as common genomic features rather than virulence factors. In addition, *W. confusa* FS54 was reported to encode a relatively high number of virulence-associated genes; however, the average number of such genes across *Weissella* species was comparable to that observed in *W. cibaria* NH9449, indicating that most *Weissella* genomes harbor a similar set of conserved VFDB-annotated genes. Importantly, these genes represent conserved physiological or regulatory functions and are not sufficient to confer pathogenicity on their own [58]. Consistent with previous *Weissella* genomic surveys, no classical virulence factors typically associated with pathogenic Gram-positive bacteria—such as secreted toxins, hemolysins, or invasion-related determinants—were identified in the Wv2365 genome. Together with the absence of hemolytic and gelatinase activities in phenotypic assays, these findings support the conclusion that the VFDB virulence-associated genes detected in Wv2365 reflect common *Weissella* and LAB genomic features rather than true or classic virulence factors.

Finally, genomic screening suggested the presence of the vancomycin resistance determinant, *vanT*-like and *vanY*-like genes, and antimicrobial susceptibility testing confirmed vancomycin non-susceptibility. *W. viridescens* PC-5 is also resistant to vancomycin [59]. Both *vanT*-like and *vanY*-like genes were found in all the sequenced *W. viridescens* genomes. Another *Weissella* species, *W. paramesenteroides*, is also known to be resistant to vancomycin [60]. Therefore, these observations support the interpretation that vancomycin resistance in *Weissella* is likely an intrinsic, chromosomally encoded trait rather than the result of recent horizontal gene transfer.

## 5. Conclusions

Genomic, metabolomic, and phenotypic analyses showed that *W. viridescens* strain Wv2365 encodes carbohydrate- and amino acid-centered metabolism and has acid/bile tolerance capacity, auto-aggregation and surface hydrophobicity, and antioxidant capacity. No acquired antimicrobial resistance was detected except vancomycin non-susceptibility which is an intrinsic chromosomally encoded trait in *Weissella*. Together, these findings support Wv2365 as a promising probiotic candidate.

## Figures and Tables

**Table 1:** Antimicrobial susceptibility of *W. viridescens* Wv2365.

Antimicrobial Agent	MIC (µg/mL) Interpretive Criteria			MIC (Wv2365)	Interpretation
	S	I	R		
Penicillin	≤8	—	—	0.38	S
Ampicillin	≤8	—	—	0.5	S
Tetracycline	≤2	4	≥8	1.5	S
Chloramphenicol	≤8	16	≥32	6	S
Vancomycin	≤2	4–8	≥16	24	R
Linezolid	≤4	—	—	2	S
Meropenem	≤1	2	≥4	0.75	S
Levofloxacin	≤2	4	≥8	1	S
Erythromycin	≤0.5	1–4	≥8	0.19	S
Clindamycin	≤0.5	1	≥2	0.25	S

MIC breakpoints were interpreted according to CLSI M45. QC testing with *S. pneumoniae* ATCC 49619 confirmed assay validity, with S (susceptible), I (intermediate), or R (resistant).

## Data Availability

All sequencing data generated in this study are publicly available. The raw sequencing reads have been deposited in the NCBI Sequence Read Archive under BioProject accession PRJNA1250212. The complete genome sequence is available in GenBank under accession number CP187404.

## Supplementary Materials

The following supporting information can be downloaded at https://www.mdpi.com/article/10.3390/biology15010063/s1, Figure S1: Pangenome of *Weissella viridescens* strains; Figure S2: Functional annotation of *W. viridescens* Wv2365 genes; Table S1: Genomic Information of *Weissella viridescens* Strains in NCBI; Table S2: Pangenome analysis of *W. viridescens*; Table S3: Single-nucleotide variants (SNVs) identified in pairwise comparisons of Wv2365 with strains XBS25 and OF45-4pH10A; Table S4: Virulence-associated genes of *W. viridescens* Wv2365; Table S5: Metabolomic profile of *W. viridescens* Wv2365. Table S6: KEGG pathway enrichment results for metabolites produced by *W. viridescens* Wv2365; Table S7: Probiotic-related genes of *W. viridescens* Wv2365 involved in environmental stress adaptation and colonization.

## Abbreviations

The following abbreviations are used in this manuscript:

LAB	lactic acid bacterium
WHO	World Health Organization
FAO	Food and Agriculture Organization
EFSA	European Food Safety Authority
CGMCC	China General Microbiological Culture Collection Center
COGs	Clusters of Orthologous Groups
GO	Gene Ontology
KEGG	Kyoto Encyclopedia of Genes and Genomes
TCA	tricarboxylic acid
DPPH	2,2-diphenyl-1-picrylhydrazyl
CAZymes	Carbohydrate-active enzymes
VFDB	Virulence Factor Database
PHI-base	Pathogen–Host Interaction database
CARD	Comprehensive Antibiotic Resistance Database
CLSI	Clinical and Laboratory Standards Institute
