# Supplementary material for: Genomic and Metabolomic Insights Into the Probiotic Potential of Weissella viridescens
Source: Biology (Basel). 2025 Dec 29;15(1):63. doi: 10.3390/biology15010063 (PMC12784881; doi:10.3390/biology15010063)
Supplement: Supplementary file 1 [file biology-15-00063-s001.zip › Supplementary Figures.pdf]

Supplementary

A

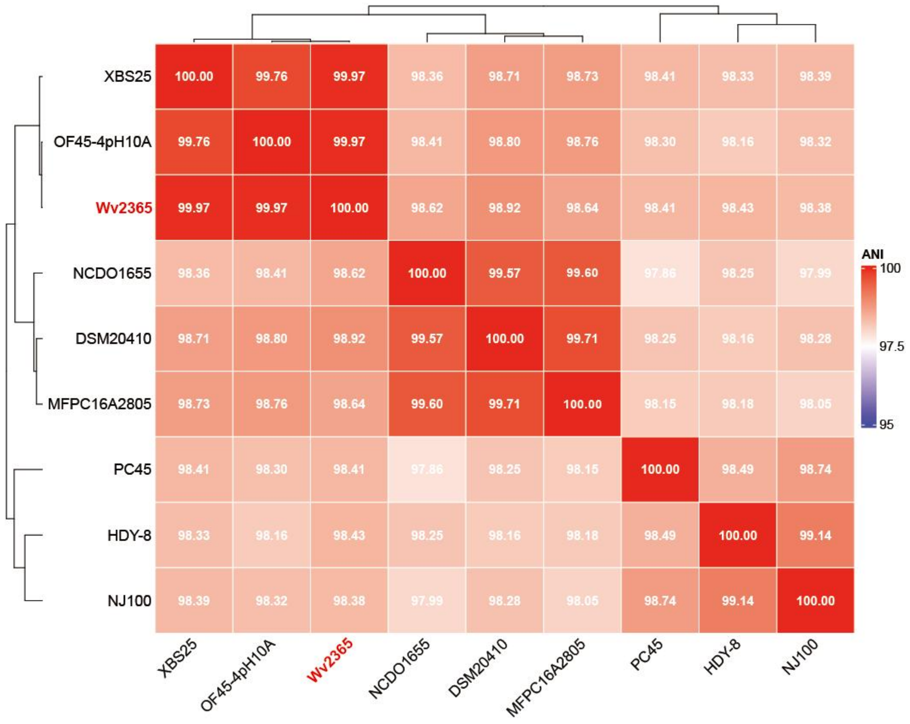

B

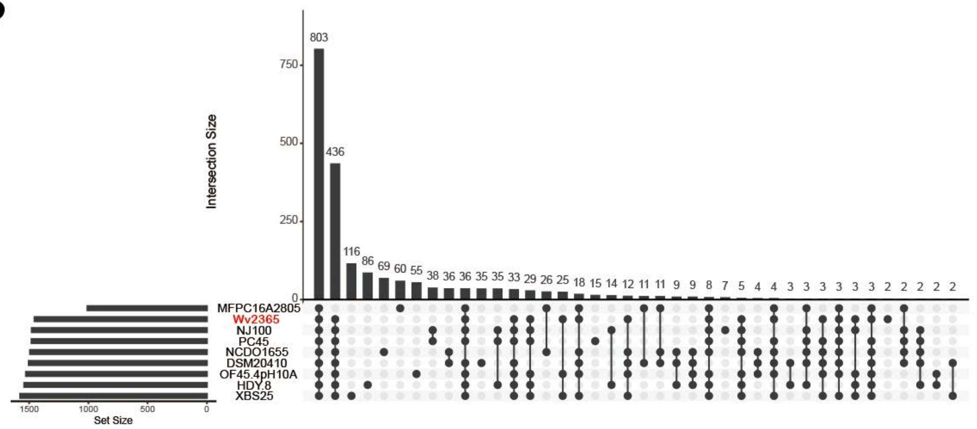

Figure S1 (A) ANI heatmap among nine *W. viridescens* genomes showing uniformly high similarity. (B) UpSet plot illustrating shared and unique gene clusters among the genomes.Set Size denotes the total number of gene clusters present in each genome, and Intersection Size indicates the number of clusters shared among the selected genomes.Wv2365 (in red) is highlighted as the focal strain to facilitate comparison with the other genomes.

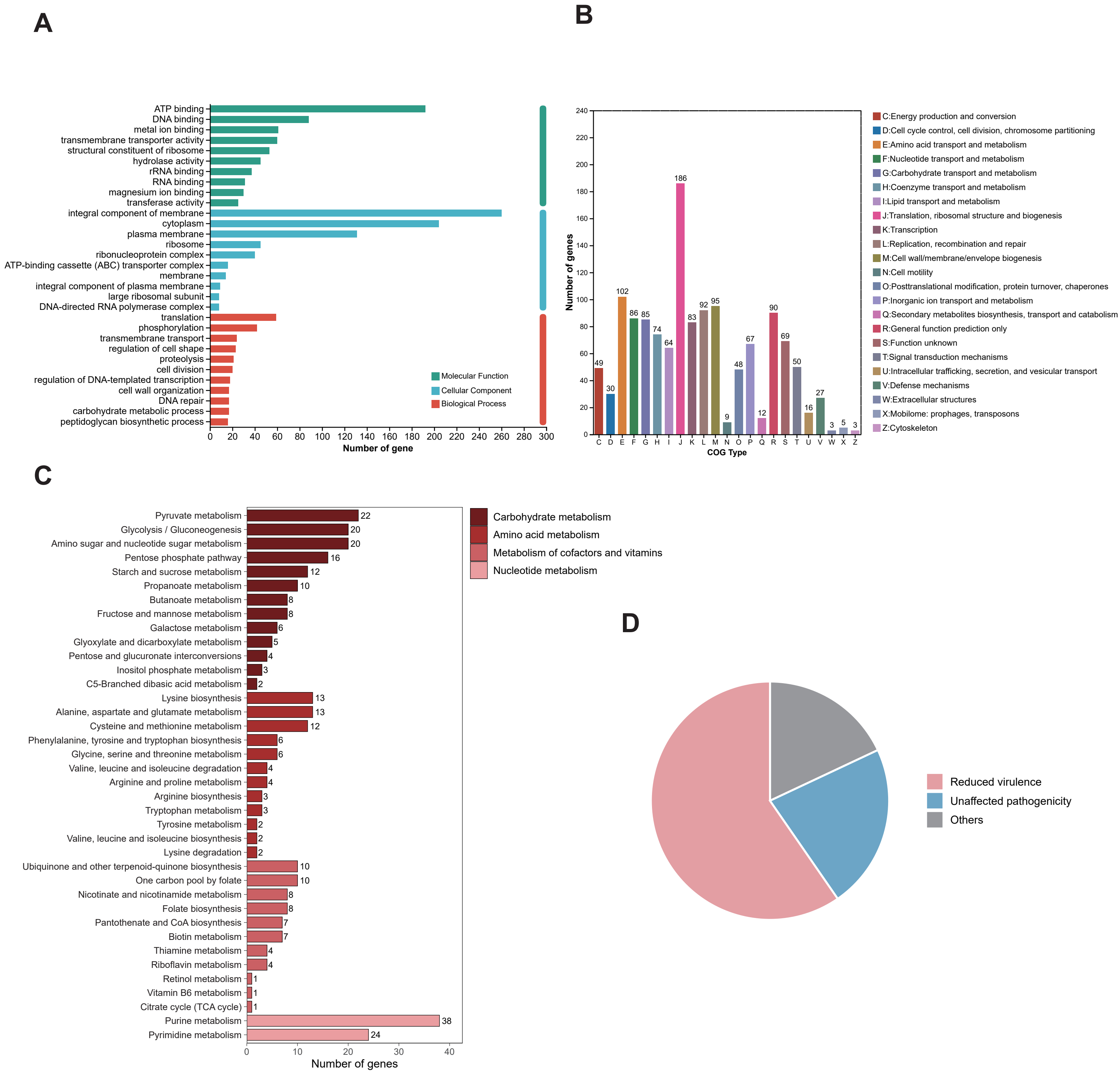

Figure S2 Functional annotation of *W. viridescens* Wv2365 genes. (A) Gene Ontology (GO) classification showing distribution across molecular function, cellular component, and biological process categories. (B) Clusters of Orthologous Groups (COG) classification illustrating the number of genes assigned to each functional category. (C) KEGG Level-3 pathways within the top4 genome categories (carbohydrate, amino-acid, cofactors/vitamins, nucleotide metabolism). (D) PHI-base phenotypic classes.
